# Hydrazones, hydrazones-based coinage metal complexes, and their biological applications

**DOI:** 10.1039/d4ra07794f

**Published:** 2025-03-03

**Authors:** Dessie Ashagrie Tafere, Mamo Gebrezgiabher, Fikre Elemo, Taju sani, Tsegaye Belege Atisme, Tesfay G. Ashebr, Ibrahim Nasser Ahmed

**Affiliations:** a Department of Industrial Chemistry, Addis Ababa Science and Technology University P.O. Box 16417 Addis Ababa Ethiopia ibrahim.nasser@aastu.edu.et tesfay.gebretsadik@aastu.edu.et; b Nanotechnology Centre of Excellence, Addis Ababa Science and Technology University P.O. Box 16417 Addis Ababa Ethiopia; c Department of Chemistry, College of Natural and Computational Science, Mekdela Amba University P.O. Box 32 Tuluawulia Ethiopia

## Abstract

Hydrazone-based compounds distinguished by their azomethine –NHN=CH group and their respective coinage metal complexes have emerged as leading candidates in the search for effective anticancer and antibiotic agents. Because of their varied pharmacological potential and simplicity of synthesis, these compounds have been the subject of substantial research. Hydrazones exhibit versatile coordination chemistry, allowing for the formation of stable complexes with metals such as copper, silver, and gold. Hydrazone derivatives and their metal complexes demonstrate significant biological activities, displaying potent anticancer properties inducing apoptosis, inhibiting cell proliferation, and disrupting angiogenesis. Furthermore, they exhibit vigorous antibiotic activity by compromising microbial cell membranes and inhibiting essential enzymes. This review article highlights the versatility and effectiveness of hydrazone-based compounds and their coinage metal complexes reported for the last five years, underscoring their potential as next-generation diagnostic and therapeutic agents. Ongoing research focuses on optimizing these compounds for more excellent selectivity, potency, and biocompatibility, which is expected to advance their practical biological applications.

## Abstract 2

Hydrazone-based compounds distinguished by their azomethine –NHN=CH group and their respective coinage metal complexes have emerged as leading candidates in the search for effective anticancer and antibiotic agents.

## Graphical abstract

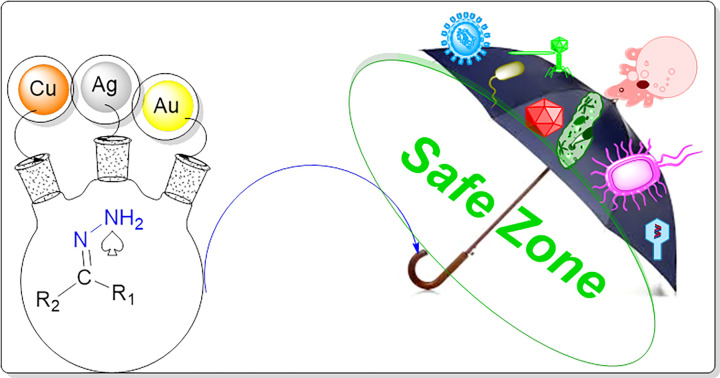

## 1. Introduction

The global rise in cancer and antibiotic-resistant infections presents a significant dual challenge to modern medicine and needs innovative therapeutic strategies.^1,2^ According to a WHO report, cancer remains a leading cause of mortality, responsible for about 1 in every six deaths and affecting nearly every household.^3^ By 2022, an estimated 20 million new cancer cases and 9.7 million deaths were reported globally, with projections indicating a 77% increase in the cancer burden by 2050, further straining health systems and communities. Simultaneously, the emergence of multidrug-resistant pathogens has severely reduced the efficiency of traditional antibiotics.^3^ Metal-based therapeutics, particularly those involving coordination compounds, have garnered significant attention to address these problems.^4^ Since the groundbreaking discovery of platinum-based anticancer properties in the 1960s, the development of metal complexes has expanded rapidly, offering new possibilities for combating cancer and infectious diseases.^5^

Hydrazones are a class of compounds featuring a carbon–nitrogen double bond adjacent to a hydrazine group, and they are exciting due to their tuneable structural and biological properties.^6^ These compounds possess functional groups such as carbonyl and imine moieties, enabling them to chelate metal ions and form stable complexes with diverse geometries.^7^ Hydrazone derivatives have demonstrated significant therapeutic potential, including antimicrobial, antioxidant, and anticancer activities.^8,9^ Notably, hydrazone-containing drugs like isoniazid (antitubercular)^10^ and nifuroxazide (antibacterial)^11^ underscore the importance of these compounds in pharmacology.

Hydrazine-based complexes, specifically those involving copper(ii), silver(i), and gold(i/iii), offer unique advantages in biomedical applications.^12^ These metals are well-known for their redox activity, biocompatibility, and ability to interact with biological targets such as enzymes, DNA, and proteins.^13,14^ Coinage metal complexes often exhibit enhanced biological activity when coordinated with hydrazone ligands, including selective cytotoxicity against cancer cells and potent antimicrobial effects against resistant pathogens.^15^

Despite the growing recognition of hydrazone and hydrazide–hydrazone compounds in medicinal chemistry, comprehensive reviews still need to address their biological activity, particularly when considering their metal complexes. Hydrazone derivatives, including hydrazide–hydrazones, are known for their ability to form stable coordination complexes with metal ions, and this coordination can significantly influence their biological properties.^16^ While early reviews, such as those by Popiolek (articles from 2010–2016), examined the biological activity of hydrazine derivatives, they did not explore the impact of metal coordination.^17^

Similarly, a review on quinoline hydrazone derivatives as a new class of potent antitubercular and anticancer agents again by Mandewale *et al.* (2017) without addressing their metal complexes.^18^ Verma *et al.* (2014) reviewed the biological activities of hydrazones but omitted a detailed exploration of their coordination with metal ions.^19^ Jabeen (2022) investigated analytical applications of hydrazone derivatives in a different context.^20^ More recently, Amadi *et al.* (2023) reviewed the synthesis, lanthanide complexes, and biological activities of hydrazone derivatives of hydrazinecarbothioamides.^21^

While previous works have extensively examined hydrazones and their metal complexes as separate entities,^16,20,22–25^ our review uniquely emphasizes the synergy between hydrazones and coinage metal ions, highlighting how metal coordination enhances their biological applications. Focusing on from 2020 to 2024, we aim to capture recent advancements and emerging trends in this rapidly evolving field, showcasing innovative approaches and applications. Despite the significant contributions made in the field, a dedicated review addressing the biological applications of hydrazone-based coinage metal complexes—particularly their anticancer and antimicrobial potential—remains lacking. This review article seeks to bridge this gap by elucidating the role of metal coordination in amplifying the therapeutic properties of hydrazone derivatives, with a particular focus on their effects against cancer and microbial infections.

## 2. Hydrazones and their derivatives

**Fig. 1:**
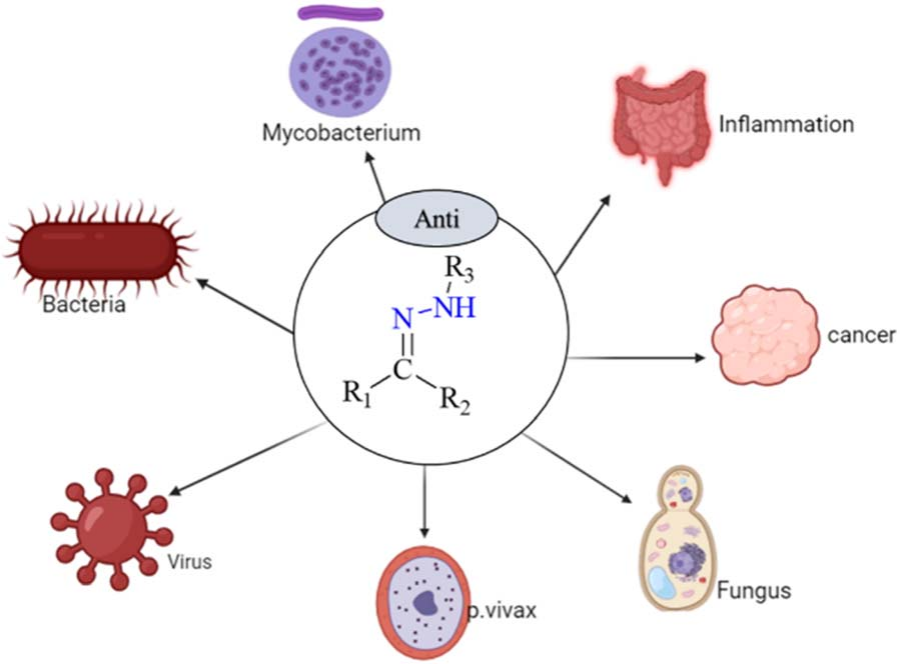
Some biological applications of hydrazone and their derivatives.^26–31^

**Scheme 1:**
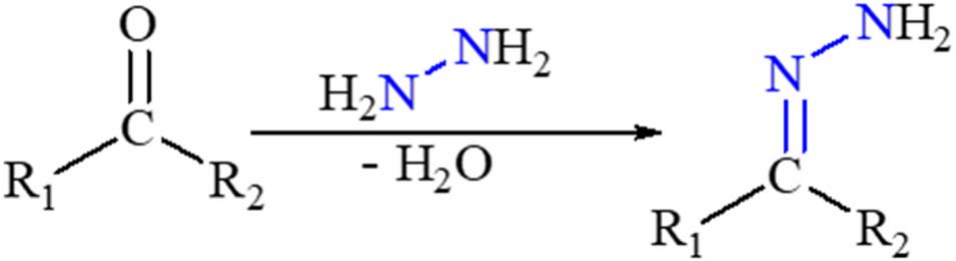
General synthesis method of hydrazones.

**Scheme 2:**
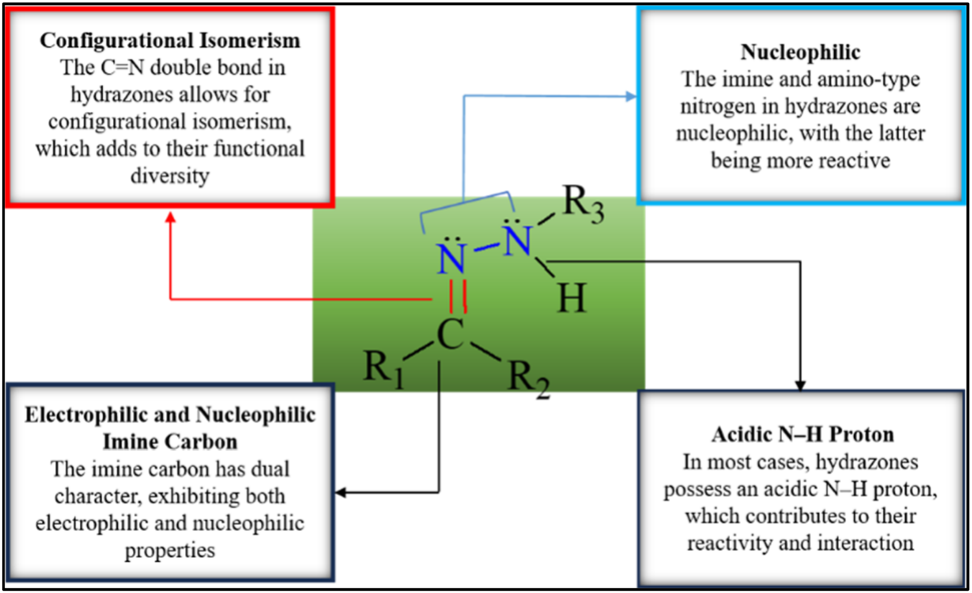
Structural and functional diversity of the hydrazine.

Numerous biological and pharmacological characteristics are possessed by these compounds, including antimicrobial,^26^ anti-inflammatory,^27^ analgesic,^27^ antifungal,^28^ anti-tubercular,^29^ antiviral,^29^ anticancer,^30^ antiplatelet,^27^ antimalarial,^31^ and anti-schistosomiasis activities (Fig. 1). Hydrazones have nucleophilic nitrogen atoms and carbon atoms that are both electrophilic and nucleophilic due to a C=N bond conjugated with a single pair of electrons on the nitrogen atom (Scheme 1). Compared to ketones, hydrazones have a more acidic α-hydrogen. Compounds with distinct physical and chemical properties can be created by combining hydrazones with various functional groups (Scheme 2). Because of their pharmacological and biological characteristics, hydrazones are considered crucial for producing heterocyclic molecules.^32,33^

### 2.1. Anticancer activities

Cancer is characterized by the rapid formation of abnormal cells that grow uncontrollably, potentially invading nearby tissues and spreading to other body parts, as defined by the World Health Organization.^34,35^ The increasing occurrence of cancer highlights the urgent need for new treatments, prompting research into both natural-inspired and synthetic approaches to identify potential anticancer metal-based drugs. One promising area of research involves the exploration of hydrazone derivatives as anticancer agents.^36^ The potential of hydrazone derivatives as anticancer medicines has been investigated in several studies.

Al-Salem *et al.* synthesized a series of *N*-benzylisatin aryl hydrazones (H_1a–j_) to evaluate their potential as anticancer agents. *N*-Benzyl isatin aryl hydrazones are synthesized by linking an isatin core with an aryl hydrazone group *via* a benzyl linker, aiming to leverage this structure for potential anticancer activity. These substances were examined in tests using the non-small cell lung cancer cell lines HeLa and A549. The outcomes showed promise, as the hydrazones showed better antiproliferative activity than gefitinib, a well-known anticancer drug for these cancer types. This suggests that *N*-benzylisatin aryl hydrazones could be a strong alternative to current treatments, highlighting their potential for further development and clinical testing.^37^

**Fig. 2:**
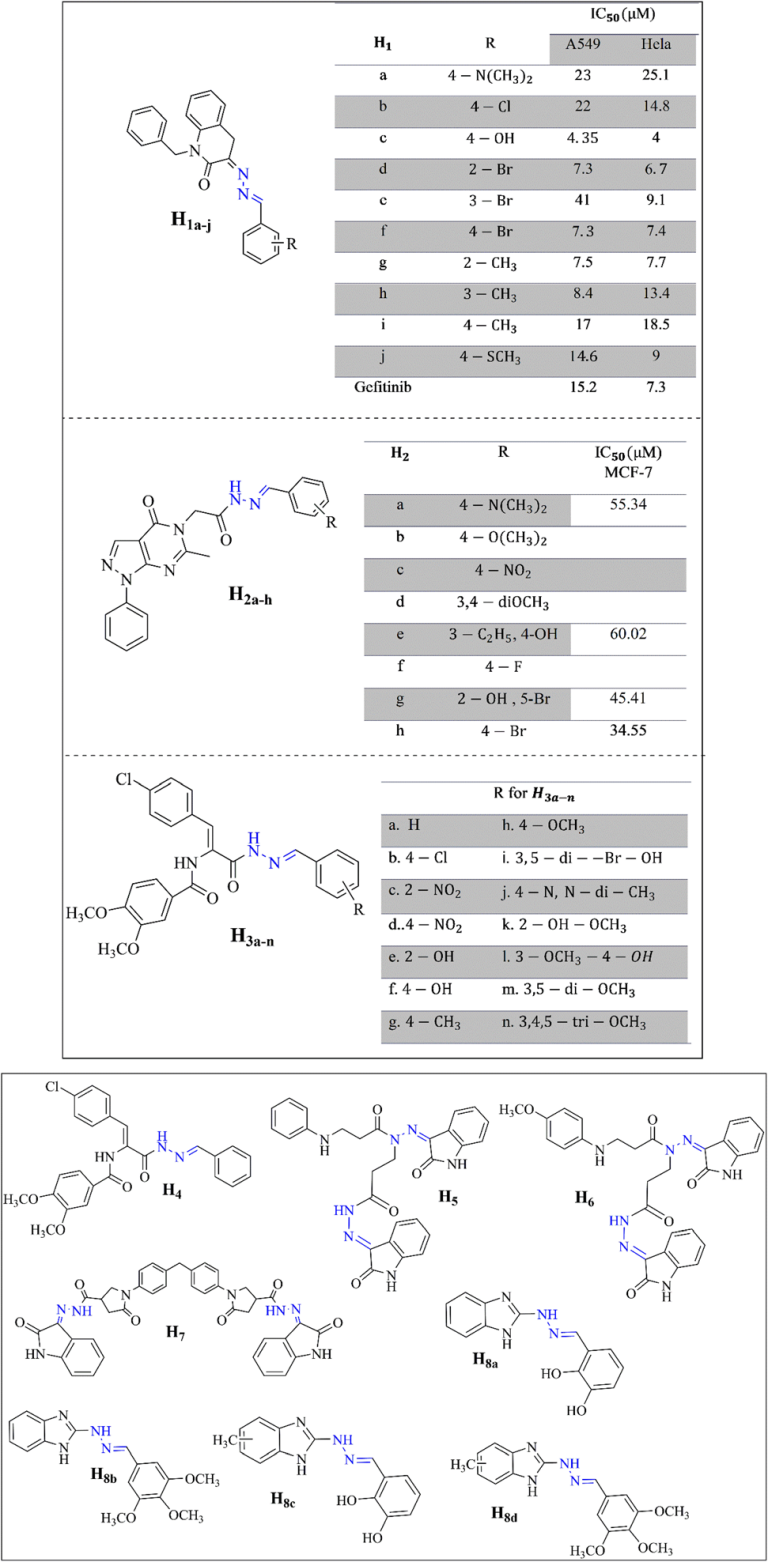
Hydrazone-based anticancer compounds (H_1_–H_8_).^37–40^

Similarly, Horchani *et al.* conducted a detailed study starting with synthesizing pyrazolopyrimidinone derivatives from 5-amino-3-methyl-1-phenyl-1*H*-pyrazole-4-carbonitrile. The research included several stages, ultimately leading to the creation of hydrazide–hydrazone derivatives (H_2a–h_). Compounds H_2a_, H_2e_, H_2g_, and H_2h_ demonstrate notable antiproliferative effects against MCF-7 breast cancer cells. These derivatives also show potential as EGFR inhibitors, a crucial target in cancer therapy, indicating their utility in developing targeted cancer treatments (Fig. 2).^38^

**Fig. 3:**
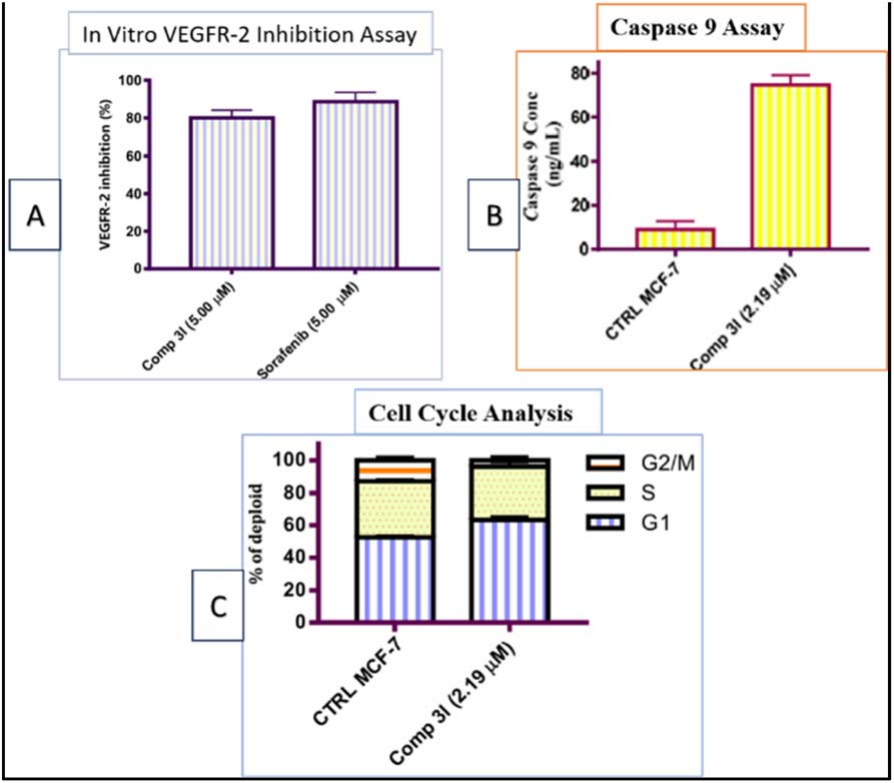
Summarized effects of 4-hydroxy-3-methoxybenzylidene hydrazinyl H_3l_ on MCF-7 cells: inhibition of VEGFR-2, suggesting anti-angiogenic properties (Panel A), alterations in cell cycle distribution (Panel B), and increased caspase 9 activation, indicating apoptosis (Panel C).^39^

Furthermore, Al-Warhi *et al.* synthesized novel hydrazone derivatives featuring a *cis*-(4-chlorostyryl) amide moiety (H_3a–h_ and H_4_). These compounds were tested for their cytotoxicity against MCF-7 breast cancer cells. Several derivatives, specifically H_3i_, H_3l_, H_3m_, and H_3n_, exhibited strong cytotoxic effects with IC_50_ values comparable to the established chemotherapeutic agent Staurosporin. Moreover, H_3l_ (Fig. 3) inhibited VEGFR-2 by 80.06% at five μM, induced G1 phase cell cycle arrest, and significantly increased active caspase nine levels, which is linked with the initiation of apoptosis.^39^

In a recent study by Tumosienė and colleagues, hydrazone-isatin derivatives were evaluated for their anticancer properties against melanoma and colon cancer cell lines. Compounds H_5_, H_6_, and H_7_ showed significant cytotoxicity, surpassing the effects of conventional chemotherapy drugs like dacarbazine and 5-fluorouracil. These derivatives effectively inhibited colony formation and reduced spheroid growth, suggesting their ability to interfere with cancer cell proliferation and viability. These results highlight the potential of hydrazone-isatin derivatives to provide more effective treatment options for melanoma and colon cancer.^8^

Yancheva *et al.* synthesized 1*H*-benzimidazol-2-yl hydrazones (H_8a–d_) that exhibit potent anticancer properties by inhibiting tubulin polymerization, with compound H_8d_ showing the most significant activity (IC_50_ = 1.2 μM). Docking studies reveal strong binding interactions with tubulin, supporting their mechanism of action. MTT (3-(4,5-dimethylthiazol-2-yl)-2,5-diphenyltetrazolium bromide) assays indicate that these compounds, particularly the methyl-substituted variants, achieve cytotoxicity with IC_50_ values ranging from 1.2 to 3.5 μM against cancer cells agents.^40^

### 2.2. Antimicrobial activity

Bacteria becoming resistant to antibiotics is a big problem, so finding new ways to fight infections is essential. The recent article by Arora *et al.* details the synthesis and characterization of four hydrazone ligands, H_9–12_, and their corresponding transition metal complexes. Various analytical techniques characterized these ligands, indicating their potential therapeutic applications.^41^ Compound H_13_, synthesized by Popiolek *et al.*, displayed notable antibacterial activity against MRSA, with a Minimal Inhibitory Concentration (MIC) of 3.91 μg mL^−1^.^42^ Meanwhile, Noshiranzadeh *et al.* synthesized lactic acid hydrazide–hydrazones, finding compounds H_14_ and H_15_ displayed notable antibacterial activity, particularly compound H_14_, attributed to its NO_2_ substituent.^43^ Helmy *et al.* also present a series of newly synthesized thiadiazolyl hydrazone derivatives (H_16–17_) with antimicrobial activity. Among the compounds tested, compound H_16_ showed activity against *E. coli*, while compound H_17_ was active against *B. mycoides*. Compound H_18_ exhibited activity against *C. albicans*. These compounds displayed inhibition zones more significant than the positive control, indicating their potential utility in inhibiting microbial spread.^44^

Tuberculosis (TB) is a chronic and infectious disease that remains highly prevalent worldwide—various strains of *Mycobacterium tuberculosis* 30 cause it. Vlad, I. M., *et al.* introduce six new NSAID-*N*-acyl hydrazone derivatives H_19–24_ with promising tuberculostatic activity against drug-resistant *Mycobacterium tuberculosis* strains.^45^

Campos *et al.* present hydrazones H_25–27_ as promising candidates for latent tuberculosis treatment, showing superior activity to isoniazid with improved safety profiles.^46^ Oderinlo*et al.* synthesized 1,2,4-triazolo-3-thiol derivatives (H_28a–g_ and H_29_) that demonstrated potential as antimycobacterial agents, exhibiting modest *in vitro* activity against *Mycobacterium tuberculosis* H37Rv with MIC_90_ values ranging from 3.99 to 12.32 μM. Compound H_29_ was the most active, with a MIC_90_ of 3.99 μM. The compounds also showed low cytotoxicity against HeLa cells, indicating a favorable safety profile.^47^

Briffotaux *et al.* identified compound H_30_ as a promising TB treatment, inhibiting MmpL3 and showing vigorous activity against *Mycobacterium tuberculosis* H37Rv and drug-resistant strains. H_30_ demonstrated MIC values of 0.2 to 0.4 μg mL^−1^ and proved non-toxic in *G. mellonella* larvae, indicating its potential for further development.^48^

Additionally, the study on aroylhydrazones, highlighting compounds H_31_ and potential in TB treatment, was conducted by Valcheva *et al.* In mice, these compounds exhibit low cytotoxicity, high selectivity, and favorable LD_50_ values (1224.7 mg kg^−1^ for H_31_ and >2000 mg kg^−1^ for H_31_) *via* intraperitoneal administration, with reduced toxicity observed orally. A 14 days subacute toxicity study reveals minimal adverse effects, suggesting their safety and efficacy.^49^

Malaria, a potentially fatal illness spread by *Plasmodium* parasites carried by infected mosquitoes, continues to pose a serious threat to global health.^50,51^ Despite progress in control measures, including vector control and chemotherapy, the emergence of drug-resistant parasite strains poses challenges to existing treatment strategies.

Magwaza *et al.* report that the 4-aminoquinoline hydrazone compounds H33 and H34 demonstrate vigorous antimalarial activity, with pIC_50_ values of 5.37 and 5.18, respectively, against the chloroquine-sensitive (CS) and chloroquine-resistant (CR) strains of *Plasmodium falciparum*.

This efficacy, coupled with low cytotoxicity and rapid onset of action, suggests their potential as novel antimalarial drugs, particularly in combination therapies to combat drug resistance.^52^ Amengor *et al.* evaluated two phenylhydrazones, H_35_ and H_36_, for their potential as antimalarial agents. Nuclear magnetic resonance confirmed these compounds showed significant antimalarial activity against chloroquine-sensitive (3D7) and resistant (DD2) *Plasmodium falciparum* strains, with pIC_50_ values of 5.37 and 5.18, respectively.^53^ Additionally, the study by Sharma *et al.* reported that compound H_37_ exhibited an IC_50_ of 0.26 μM against a chloroquine-resistant *Plasmodium falciparum* strain. Mechanistic insights suggest its potential inhibition of hemozoin formation, with binding affinity to heme comparable to chloroquine (Fig. 8).^54^

Hydrazones are also used in sonodynamic therapy to generate singlet oxygen and target cancer cells under ultrasound irradiation. Li *et al.* recently reported the synthesis and application of a novel zinc(ii) complex, ZnAMTC, aimed at enhancing sonodynamic therapy (SDT) efficacy against tumors (Fig. 9A). The complex, derived from a hydrazone ligand, exhibited remarkable properties in their study. Under ultrasound (US) irradiation, ZnAMTC demonstrated the efficient generation of singlet oxygen (^1^O_2_), essential for SDT, with a quantum yield of *ΦΔ* = 0.72. Notably, the complex showed minimal dark toxicity and potent cytotoxicity against a range of cancer cells, with IC_50_ values in the low micromolar range. Mechanistic investigations revealed that ZnAMTC functions by reducing intracellular glutathione (GSH) levels and inhibiting glutathione peroxidase 4 (GPX4) activity upon US exposure, thus inducing ferroptosis in cancer cells (Fig. 9B). *In vivo* studies further validated ZnAMTC's efficacy, demonstrating significant tumor growth inhibition under US irradiation while maintaining good biosafety profiles (Fig. 9C). This research highlights the promising role of first-row transition metal complexes like ZnAMTC in advancing SDT strategies through efficient design and synthesis approaches.^55^

## 3. Biological applications of hydrazone-based coinage metal complexes

Coinage metals copper, silver, and gold have long been valued for their economic significance and diverse functional applications. Historically, these metals were prized for their use in coinage, a testament to their durability, malleability, and luster.^56^ However, due to their unique physical and chemical properties, their utility extends far beyond monetary value, playing critical roles in various scientific and technological fields. For instance, copper's high electrical conductivity makes it indispensable in wiring and electronics,^57^ while gold's resistance to corrosion is crucial in high-performance electronic devices and reliable connectors.^58^ Additionally, these metals are exceptional thermal conductors and effective catalysts, enhancing numerous industrial processes.^59^

The intersection of coinage metals with biology has opened new avenues for medical and therapeutic innovations. The chemical versatility of these metals, particularly their ability to form stable complexes with various ligands, underpins their biomedical potential.^60^ Hydrazones, a class of biologically active ligands, often form stable complexes with these metals, enhancing their biological activity and specificity.^61,62^ Research into these metal–ligand complexes has revealed promising results in cancer therapy, where they exhibit significant cytotoxic effects against cancer cells and as antimicrobial agents, addressing the growing issue of antibiotic resistance.

### 3.1. Copper complexes

**Scheme 3:**
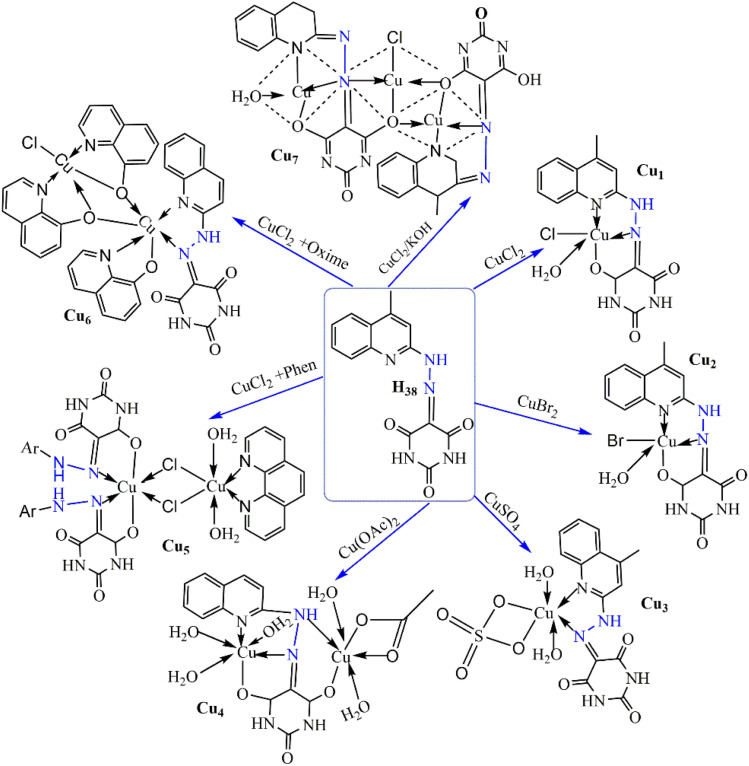
Synthesis of antitumor copper(ii) complexes (Cu1–Cu7) with H38.^63^

El-Inany *et al.* reported on testing H_38_ and its copper(ii) complexes (Cu_1_–Cu_7_) (Scheme 3) for their antitumor activity against Ehrlich Ascites Carcinoma. While AlloxHQ exhibited modest activity (IC_50_ = 112.8 μg mL^−1^), all copper(ii) complexes showed improved efficacy (IC_50_ = 41.3–60.4 μg mL^−1^) (Fig. 10). Enhanced activity in the copper(ii)–AlloxHQ complexes may result from expanded conjugation due to copper coordination. The nature of the counter anion also influenced activity, with sulfate > bromo > chloro > acetate complexes. The mechanism of action is complex, possibly involving interaction with nucleoside bases or metal ions and H-bonding interference with cell functions.^63^

The study by Elsayed and his coworkers evaluates copper(ii) hydrazone complexes (Cu_8_ and Cu_9_), Cu_9_ exhibited potent cytotoxicity against HCT116 and MCF7 cells, with IC_50_ values of 30.64 ± 2.5 μM and 37.56 ± 2.6 μM after 24 hours, and 27.03 ± 2.1 μM and 31.65 ± 2.6 μM after 48 hours, respectively. Its therapeutic index (TI) was 2.12 after 24 hours, 2.81 after 48 hours for HCT116 cells, 1.72 after 24 hours, and 2.40 after 48 hours for MCF7 cells.^64^

The biological application of copper complexes Cu_10a–b_, as elucidated by Thirunavukkarasu *et al.*, showcases their significant antiproliferative activity against various cancer cell lines, including pancreatic PSN-1 cells, where they were found to be 3-fold more effective than cisplatin, with IC_50_ values of 3.9 ± 0.3 μM and 4.7 ± 0.8 μM, respectively. Moreover, both complexes demonstrated the ability to overcome multidrug and oxaliplatin resistance in ovarian and colon cancer cells, with IC_50_ values ranging from 2.3 ± 0.7 μM to 4.6 ± 0.6 μM.^65^

The innovative use of pharmaceutically active molecules as HH ligands, as explored by Kaur *et al.*, involved synthesizing hydrazone derivatives by combining pyridine and imidazole aldehydes with NSAIDs, followed by examining their copper(ii) complexes. These complexes exhibited a square planar shape, with ligands functioning as bidentate in diclofenac hydrazone complexes and tridentate in ibuprofen–hydrazone conjugates. The MDA-MB-231 triple-negative breast cancer cell line had the lowest IC_50_ values (3.4–6.6 μM), indicating modest cytotoxic activity against the A549 and HTC-116 cancer cell lines. Ibuprofen-imidazole hydrazone combination and its Cu(ii) complex demonstrated the most notable anticancer activity, specifically Cu_11_.^66^

Chennam *et al.* synthesized the copper(ii) compound Cu_12_. This combination exhibited radical scavenging action and the ability to bind bovine serum albumin (BSA) and operate as a DNA intercalator. Additionally, it exhibited anti-proliferative effects on the cancer cell lines HeLa and MCF-7, causing morphological alterations such as nuclear enlargement, cytoplasmic blebbing, and late apoptosis, with IC_50_ values of 26 μM and 42 μM, respectively.^67^

By condensation of α-tetralone with several hydrazide derivatives (nicotinic acid, benzoic acid, *p*-toluic acid, and isonicotinic acid), Devi *et al.* produced hydrazone–hydrazone (HH) molecules. They assessed their Cu(ii) complexes. The most active compounds were those containing methyl benzoic and nicotinic hydrazones Cu_13_. Additionally, these substances demonstrated selectivity for cancer cells, showing a marked decrease in toxicity toward normal rat skeletal myoblast L6 cells.^62^ In a follow-up study in 2022, Devi *et al.* synthesized similar metal complexes using 6-chlorothiochroman-4-one instead of α-tetralone. The Cu(ii) complex derived from nicotinic hydrazide Cu_14_ exhibited the most potent cytotoxicity, with low micromolar activity against A549, DU145, and SW620 cancer cell lines, inducing reactive oxygen species (ROS) generation and apoptosis through mitochondrial depolarization.^68^

Halogen atoms—fluorine in particular—are frequently used in pharmaceutical medication creation. To achieve high anticancer activity in Bel-7402, HeLa, MCF-7, and MGC-803 cell lines (IC_50_ ≤ 5 μM), Jiang *et al.* synthesized dinuclear copper(ii) complexes with halogen-substituted 2-hydroxybenzylidene benzohydrazides. These complexes also demonstrated an anti-proliferative effect on normal lung fibroblasts (WI-38). The fluorine-substituted compound Cu_15_ caused mitochondria-mediated death in HeLa cells. It is bound to human serum albumin (HSA) in the hydrophobic cavity of a subdomain, exhibiting a cytotoxicity order of F > Cl > Br.^69^ Similarly, Kavitha *et al.* synthesized a Schiff base, 4-fluoro-*N*-((3-hydroxy-5-(hydroxymethyl)-2-methylpyridin-4-yl)methylene)benzohydrazide, and its Cu_16_. This complex intercalated with calf thymus DNA (ctDNA) and cleaved supercoiled pBR322 DNA to its nicked form without any oxidant, demonstrating fluorine incorporation's efficacy in enhancing anticancer properties.^70^

**Fig. 4:**
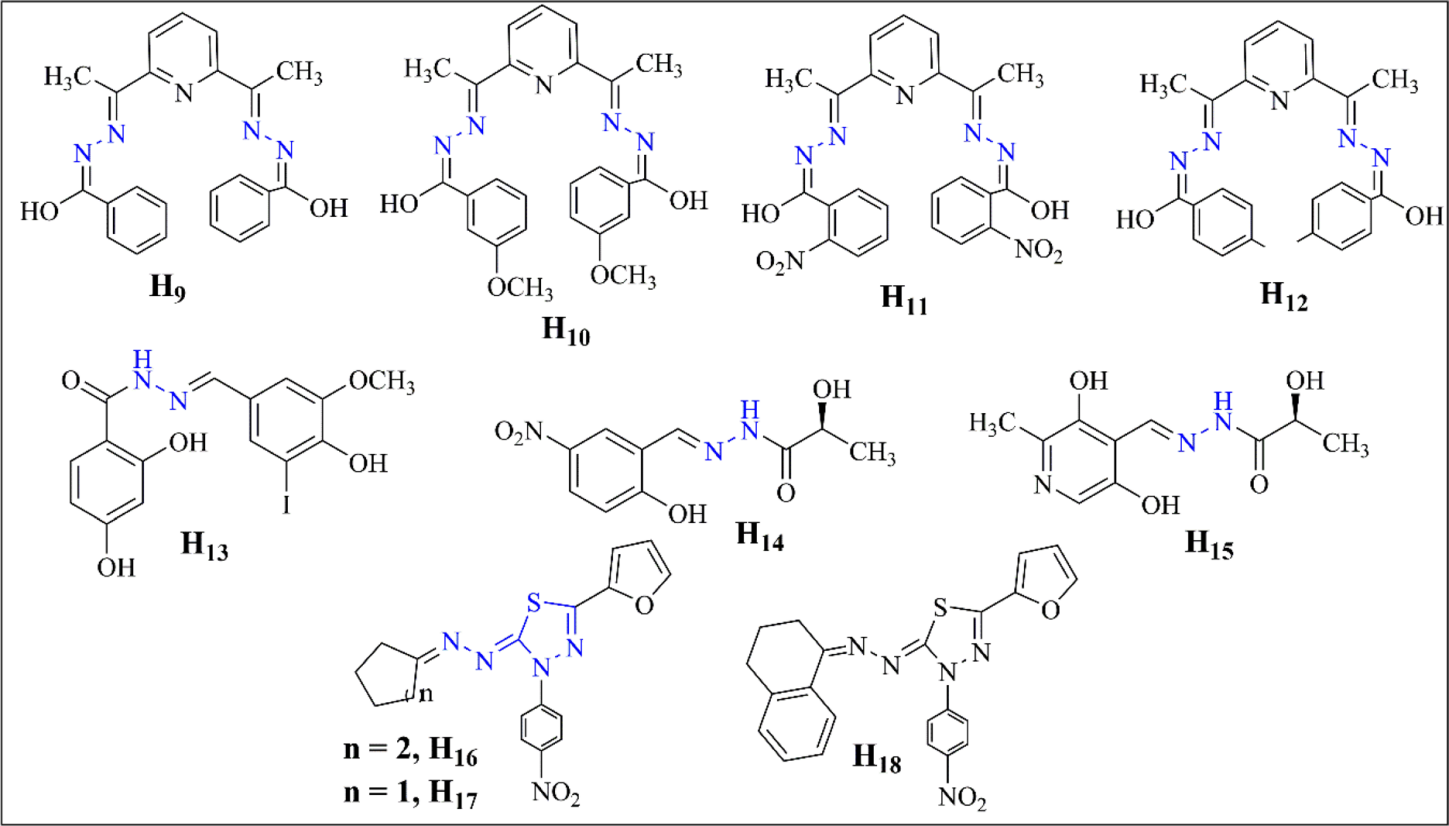
Some hydrazone-based antibacterial compounds (H_9_–H_18_).^41–44^

Burgos-Lopez *et al.* synthesized a copper(ii) complex with a tridentate *N*-acylhydrazone from 4-hydroxybenzohydrazide and 2-hydroxy-3-methoxybenzaldehyde Cu_17_, showing anticancer activity in various cell lines (IC_50_ ≤ 12 μM).^71^ Later, they improved its cytotoxicity by adding bipy or phen as co-ligands, with the phen-containing complex Cu_18_, (Fig. 4) showing lower IC_50_ values.^72^ Balsa *et al.* demonstrated this potential by synthesizing a similar complex with 4-methoxybenzohydrazide Cu_19_, which showed lower IC_50_ values than cisplatin and affected both 2D and 3D breast cancer cell cultures. However, it lacked selectivity, impacting non-tumoral cells as well.^73^

**Fig. 5:**
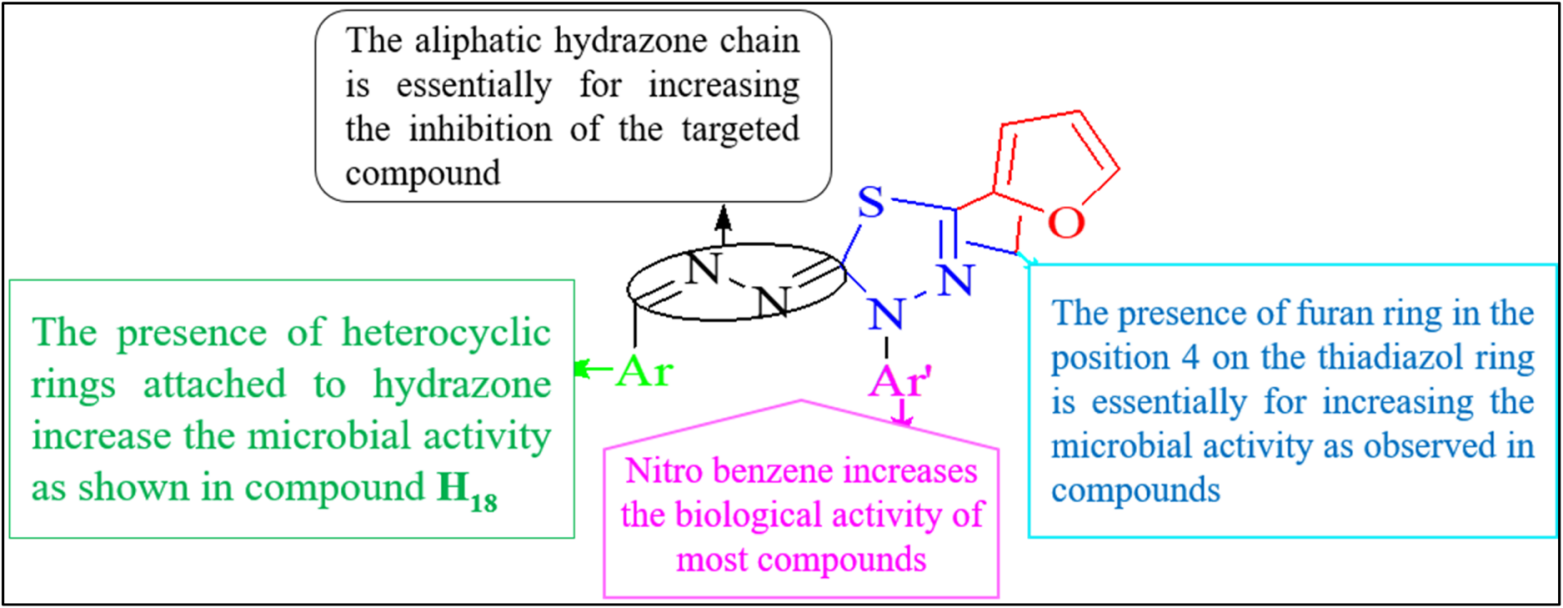
Structure–activity relationship (SAR) of (H_16_–H_18_).^44^

Numerous research groups have thoroughly investigated producing hydrazide–hydrazone complexes by employing *N*-heterocycles as co-ligands. In MCF-7, HepG2, and HCT116 (colon) cancer cell lines, Elsayed *et al.* recently discovered copper(ii) complexes of dehydroacetic acid benzoyl hydrazone with imidazole and pyridine co-ligands Cu_20_, showing better cytotoxicity compared to cisplatin with IC_50_ values about 30 μM. These complexes caused cell cycle arrest in the G2/M phase, apoptosis, and DNA fragmentation (Fig. 5).^74^

Meanwhile, Abdelrahman *et al.* explored larger aromatic systems, synthesizing a copper(ii) complex Cu_21_ with 5,6-diphenyl-4-carboxylicacid[1-(2-hydroxy naphthalene)methylidene]-hydrazide-3(2*H*)—pyridazine. This compound showed comparable IC_50_ values to cisplatin against Hep-G2 cells, targeting VEGFR-2 and triggering DNA damage and programmed cell death.^75^

Wu *et al.* also synthesized a copper(ii) complex Cu_22_ with furanyl and pyridine co-ligands by utilizing the naphthoyl moiety in ligands with hydrazide–hydrazones bearing distinct substituents. The complex demonstrated anti-metastatic properties, cytotoxicity towards A549 lung cancer cells, and induction of mitochondria-mediated apoptotic cell death with cell cycle arrest at the S phase. These findings underscore the possibility of combining naphthol with aromatic heterocyclic rings for various biological effects.^76^

Santiago *et al.* conducted an antibacterial investigation and found that the copper(ii) complex Cu_23_ has substantial antimicrobial activity against *S. epidermidis*, *E. faecalis*, *S. aureus*, and *C. neoformans*, with minimum inhibitory concentrations (MIC) of 8 μM. In addition to molecular docking experiments showing increased fitness and binding energy compared to the free ligand, indicating enhanced activity, the complex demonstrated improved efficacy against Gram-positive bacteria. Crucial interactions with active site residues, such as Val23 in *E. faecalis* and His81 in *E. aerogenes*, supported its antimicrobial potential.^77^

**Fig. 6:**
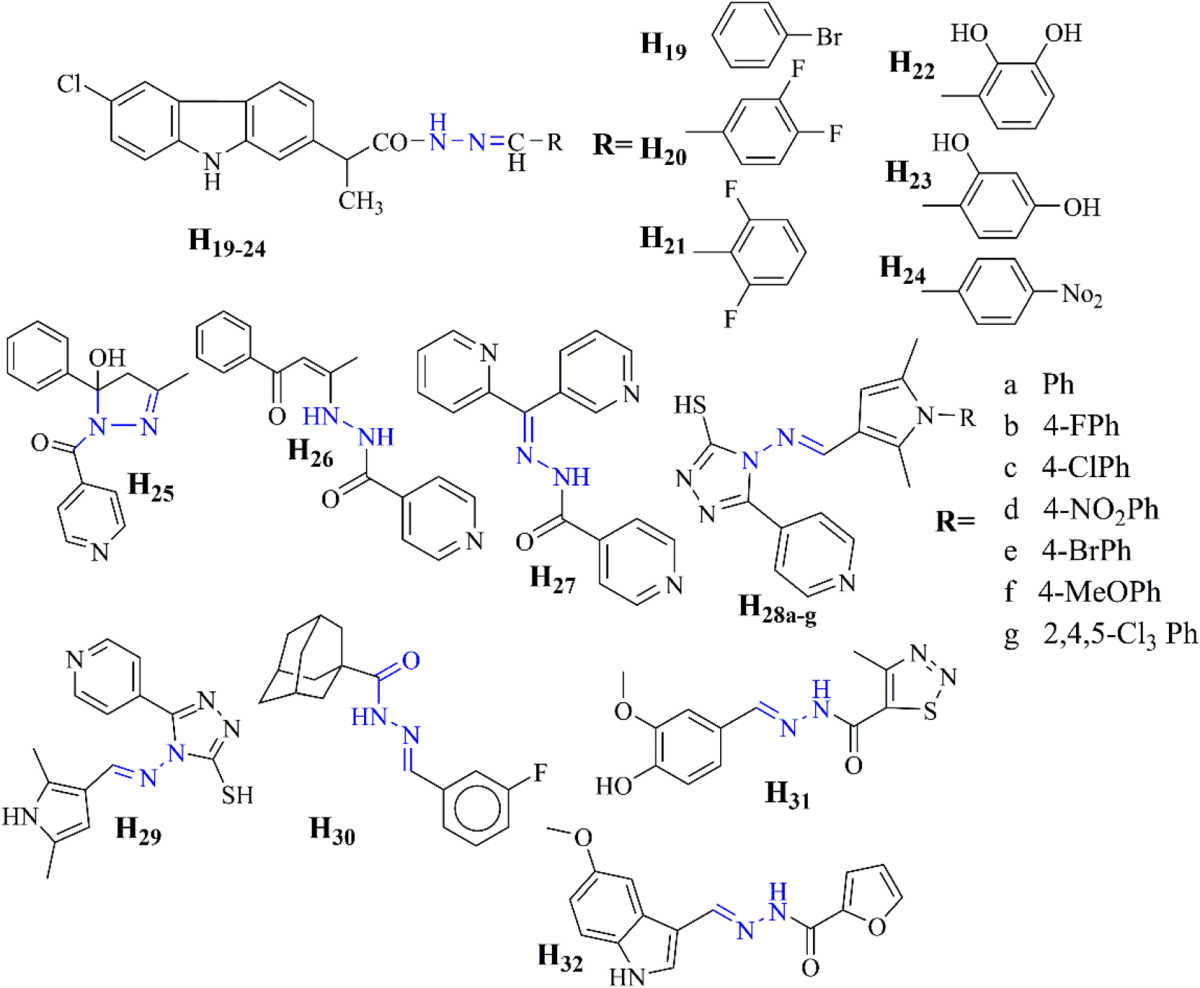
Hydrazone-based antituberculosis compounds (H_19_–H_32_).^45–49^

As reported by Kumar *et al.*, (Cu_24_) demonstrated remarkable efficacy against both TB and microbial strains. It showed potency comparable to standard drugs with a MIC value of 0.0130 μmol mL^−1^ for anti-TB activity and a MIC value of 0.0260 μmol mL^−1^ for antimicrobial activity (Fig. 6).

**Fig. 7:**
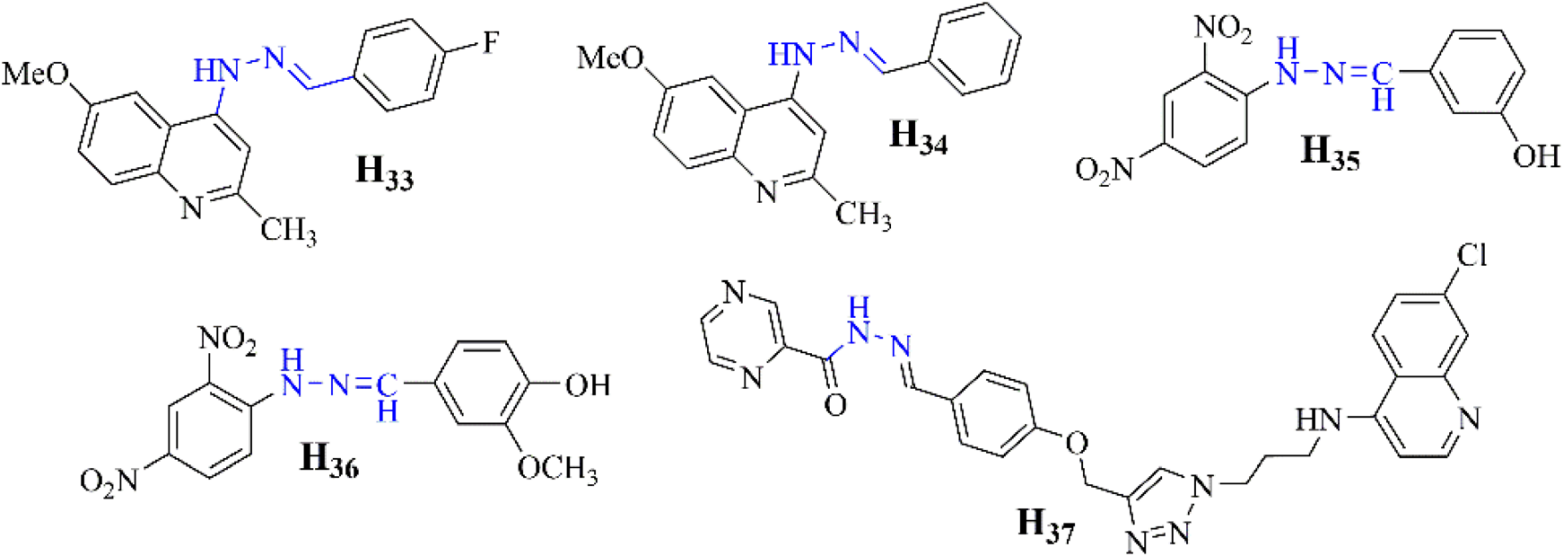
Hydrazone based antimalaria compounds (H_33_–H_37_).^52–54^

This complex presents a promising avenue for addressing TB and microbial infections, suggesting its potential for further therapeutic development.^78^ Additionally, copper(ii) complexes Cu_25_ and Cu_26_ were evaluated against various bacterial and fungal strains by Hussain *et al.*, While copper(ii) complex Cu_25_ showed inhibition zones of 10 mm against *Salmonella*, 11 mm against *Escherichia coli*, 7 mm against *Bacillus halodurans*, and 13 mm against *Aspergillus flavus*, complex Cu_26_ exhibited inhibition zones of 14 mm against *Escherichia coli*, 13 mm against *Bacillus halodurans*, and 6 mm against *Aspergillus niger*. These findings highlight copper(ii) complexes' potential in combating bacterial and fungal infections (Fig. 7).^79^

**Fig. 8:**
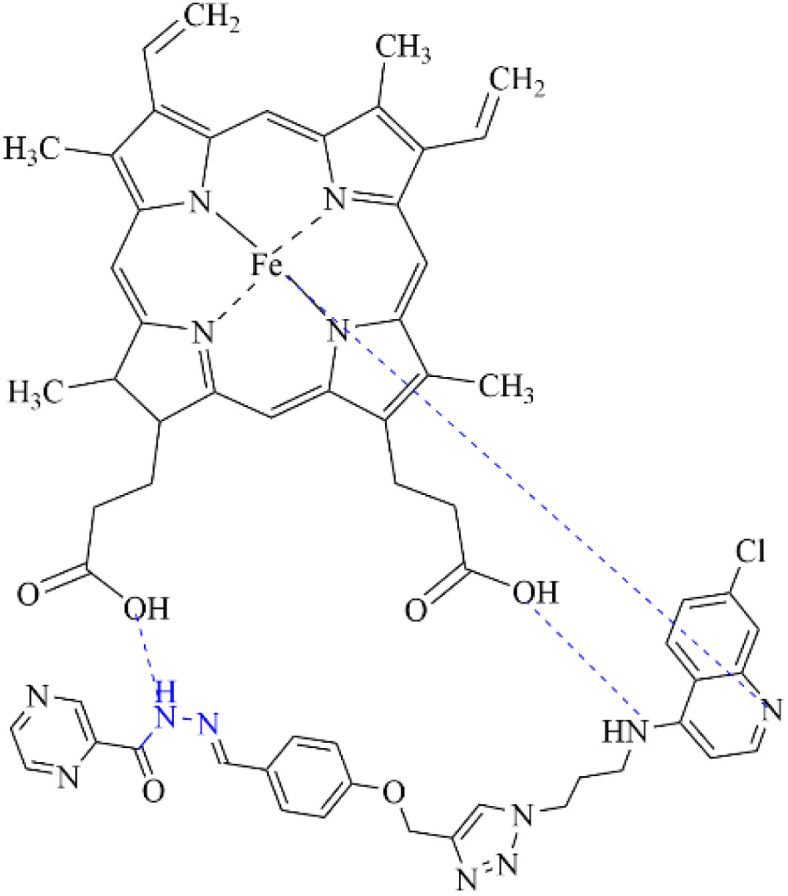
Proposed binding of heme with H_37_.^54^

**Fig. 9:**
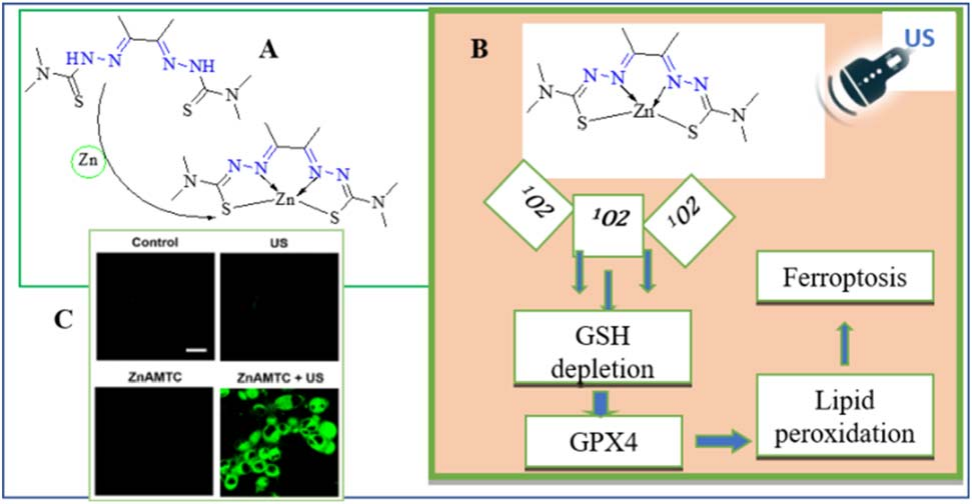
(A) Synthetic routine of for the preparation of ZnAMTC. (B) The SDT process's ferroptosis mechanism. (C) A picture depicting a fluorescence nature of C11-BODIPY-stained 4T1 cells following various therapies. C11-BODIPY: 1.0 MHz, 3 W cm^−2^, 10% duty cycle, 20 min; λ_ex_ = 488 nm; λ_em_ = 570 ± 50 nm; scale bar: 20 μm.^55^

**Fig. 10:**
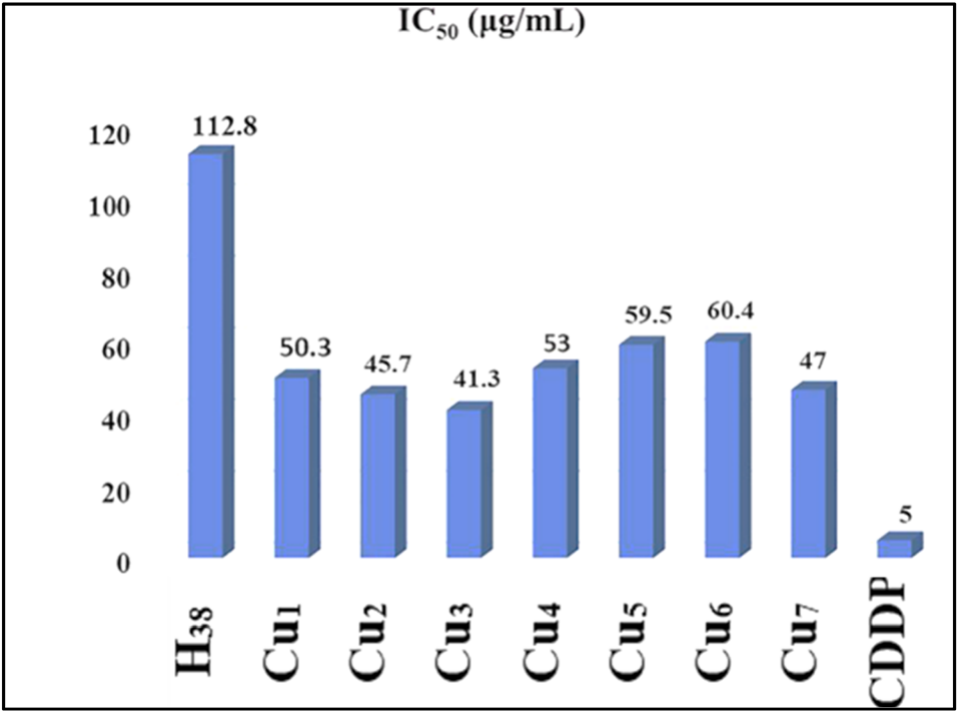
Antitumor activity of H_38_ and (Cu_1_–Cu_7_) complexes on Ehrlich Ascites Carcinoma cell line.^63^

According to Ekennia *et al.*, the copper(ii) complex Cu_27_ demonstrates strong DNA binding (*K*_b_ = 1.4 × 10^5^ M^−1^), efficient DNA cleavage at 10 μM with peroxide, and notable antimicrobial activity, especially against *Staphylococcus aureus* (MIC = 3.90 μg mL^−1^). Molecular docking studies confirm its significant interactions with DNA (binding energy = −8.9 kcal mol^−1^) (Table 1).^81^

**Table 1:** Summarized antitumor activity of copper complexes

Complex	Target cell line	IC_50_ (μg mL^−1^*) or (μM)	Reference
Cu_1_	Ehrlich Ascites Carcinoma	50.3*	63
Cu_2_		45.7*	
Cu_3_		41.3*	
Cu_4_		53.0*	
Cu_5_		59.5*	
Cu_6_		60.4*	
Cu_7_		47.0*	
Cu_8_	HCT116, MCF7	30.64, 37.56	64
Cu_9_	HCT116, MCF7	27.03, 31.65	
Cu_10a_	PSN-1	3.9	65
Cu_10b_	PSN-1	4.7	
Cu_11_	MDA-MB-231	3.4–6.6	66
Cu_12_	HeLa, MCF-7	26, 42	67
Cu_13_	A549, DU145, SW620	Low	62
Cu_14_	A549, DU145, SW620	Low	68
Cu_15_	HeLa	≤5	69
Cu_17_	Various cell lines	≤12	71
Cu_20_	MCF-7, HepG2, HCT116	∼30	74
Cu_21_	Hep-G2	Comparable to cisplatin	75
Cu_29a_	MCF-7	1.90*	80

The findings, presented by Gatto *et al.*, demonstrate that the synthesized compounds, including free ligands and copper(ii) complexes Cu_28a–d_, exhibited vigorous antibacterial activity against both *Staphylococcus aureus* and *Escherichia coli*, with minimum inhibitory concentration (MIC) values as low as 1.37 × 10^−6^ μg mL^−1^ for *S. aureus* and 1.73 × 10^−6^ μg mL^−1^ for *E. coli*. Molecular docking simulations showed strong binding affinity with scores of −8.0 kcal mol^−1^ for *S. aureus* and −7.9 kcal mol^−1^ for *E. coli*. These findings suggest the compounds could be promising candidates for new antibacterial treatments, especially for infections resistant to conventional antibiotics (Table 2).^82^

**Table 2:** Summarized antimicrobial activity of copper complexes

Complex	Target organism	Activity measure	Ref.
Cu_23_	*S. epidermidis*, *E. faecalis*, *S. aureus*, *C. neoformans*	MIC = 8 μM	77
Cu_24_	TB and microbial strains	MIC = 0.0130 μmol mL^−1^ (TB), 0.0260 μmol mL^−1^ (antimicrobial)	78
Cu_25_	*Salmonella*, *E. coli*, *B. halodurans*, *A. flavus*	10 mm, 11 m, 7 mm, 13 mm	79
Cu_26_	*E. coli*, *B. halodurans*, *A. niger*	14 mm, 13 mm, 6 mm	
Cu_27_	*S. aureus*	MIC = 3.90 μg mL^−1^	81
Cu_28a–d_	*S. aureus*, *E. coli*	MIC = 1.37 × 10^−6^ μg mL^−1^ (*S. aureus*), 1.73 × 10^−6^ μg mL^−1^ (*E. coli*)	82
Cu_29a_	*E. coli*, *P. vulgaris*,*B. subtilis*,*S. aureus*,*A. fumigatus*,*C. albicans*	40 mm, 35 mm, 32 mm, 35 mm, 41 mm, 37 mm	80

The copper(ii) complexes, Cu_29a–b_, synthesized by Elsayed *et al.*, demonstrate potent antimicrobial and antitumor activity Cu_29a_ displays superior inhibition zones (40 ± 0.23 mm for *E. coli*, 35 ± 0.26 mm for *P. vulgaris*, 32 ± 0.36 mm for *B. subtilis*, 35 ± 0.41 mm for *S. aureus*, 41 ± 0.37 mm for *A. fumigatus*, and 37 ± 0.09 mm for *C. albicans*) and an IC_50_ value of 1.90 ± 0.1 μg mL^−1^ against breast carcinoma cell lines (MCF-7), indicating promising anticancer potential. Additionally, strong DNA binding is observed, with Cu_29a_ exhibiting the highest binding affinity (binding constant: 4.75 × 10^4^). Molecular docking simulations suggest high affinity to key protein targets, highlighting their potential as inhibitors against diseases like COVID-19 and cancer.^80^

In summary copper(ii) complexes have shown promising antitumor and antimicrobial activities in various studies. For example, copper(ii)–hydrazone complexes, like Cu9, exhibited potent cytotoxicity against cancer cell lines such as HCT116 and MCF7, with favourable therapeutic indices. These complexes have demonstrated their ability to overcome drug resistance, particularly in pancreatic and ovarian cancer cells, suggesting their potential as alternatives to conventional chemotherapies. Furthermore, copper(ii) complexes like Cu23 and Cu24 displayed significant antimicrobial activity against various bacteria and fungi, including strains resistant to traditional drugs, highlighting their potential in combating multidrug-resistant infections. The interactions of these complexes with DNA, their ability to induce apoptosis through generating reactive oxygen species (ROS), and their antimicrobial efficacy underscore their potential as therapeutic agents. Additionally, including specific ligands, such as halogenated or imidazole derivatives, enhances their anticancer and antimicrobial properties, making them valuable candidates for future drug development. The ability of these complexes to target multiple pathways, including enzyme inhibition and DNA binding, opens up new avenues for treating a range of diseases, including cancer and infectious disease.

### 3.2. Gold complexes

**Fig. 11:**
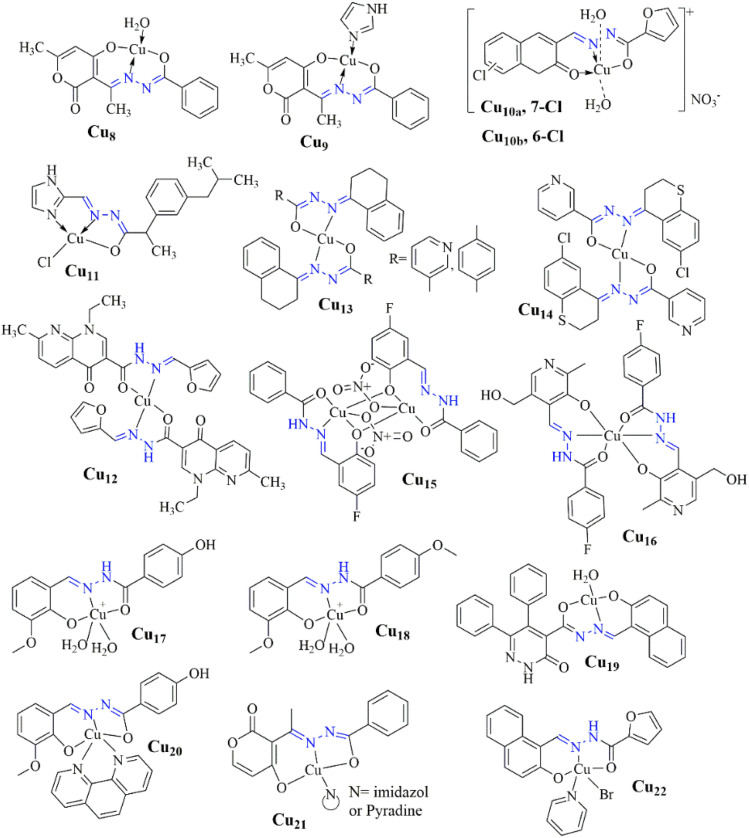
Structure of anticancer copper(ii) complexes (Cu_8_–Cu_22_).^64^

In the study by Al-Radadi and El-Gamil, newly synthesized gold(iii) complexes (Au_1_ and Au_2_) were evaluated for their anticancer effects against human cervical (prostate) and mammary gland (MCF-7) carcinoma cell lines using the MTT assay. The IC_50_ values for Au_1_ were 105.1723 μg mL^−1^ for MCF-7 and >200 μg mL^−1^ for prostate, while for Au_2_, the values were 185.1478 μg mL^−1^ for MCF-7 and 41.6551 μg mL^−1^ for prostate. These findings indicate potential anticancer activity, particularly for Au_2_ against prostate cancer (Fig. 11).^83^

**Fig. 12:**
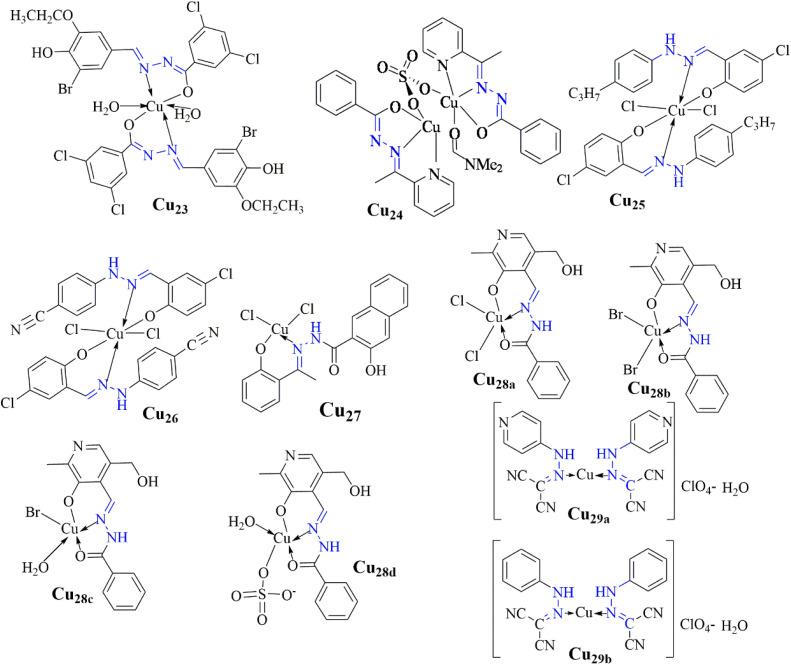
Structure of antimicrobial copper(ii) complexes (Cu_23_–Cu_29_).^77–81^

The synthesized gold(iii) complexes (Au_3a–d_), as detailed by Kanthecha *et al.*, have demonstrated significant potential in diverse biological applications. They exhibit notable cytotoxicity against brine shrimp, with LC50 values ranging from 7.1 to 26.2 μg mL^−1^, highlighting their efficacy as cytotoxic agents. Moreover, *in vivo* studies on *Schizosaccharomyces pombe* cells indicate cytotoxicity levels between 60% and 95%, with Au_3a_ exhibiting the highest potency. Additionally, these complexes show promising antibacterial activity against various strains, with MIC values ranging from 68 to 340 μM, surpassing those of the ligands and comparable to standard drugs like gatifloxacin (GFLH) and norfloxacin (NFLH). Furthermore, DNA cleavage studies reveal concentration-dependent genotoxicity, suggesting their potential to disrupt DNA integrity (Fig. 12).^84^

**Fig. 13:**
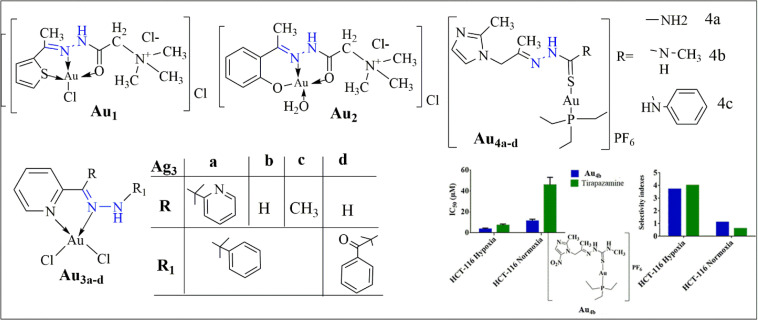
Structure of some gold complexes (Au_1_–Au_4_).^83–85^

The study conducted by Oliveira *et al.* explores the biological application of triethylphosphinegold(i) complexes with secnidazole-derived thiosemicarbazones (Au_4a–c_), focusing on their cytotoxic effects against cancer cells. The research highlights complex Au_4b_ significant hypoxia-selective cytotoxicity against colorectal cancer cells (HCT-116), exhibiting an IC_50_ value of 3.5 ± 0.9 μM under hypoxia compared to 11.3 ± 1.7 μM under normoxia, resulting in a selectivity index (SI) of 3.7, akin to the control drug tirapazamine (SI = 4) Fig. 13. These numerical values underscore complex potential as a targeted anticancer agent, suggesting its efficacy in treating hypoxic tumor regions that are typically resistant to conventional therapies.^85^

In summary, gold complexes, particularly gold(iii) and gold(i) derivatives, exhibit distinct coordination behaviours based on the oxidation state of gold and the nature of the hydrazone ligands. Gold(iii) complexes typically adopt octahedral or square planar geometries, with hydrazone ligands coordinating through nitrogen and oxygen donors, influencing their biological properties. In contrast, gold(i) complexes, often with triethylphosphine ligands, show linear coordination involving nitrogen atoms of the hydrazones. These coordination characteristics contribute to their anticancer activity, with some gold(iii) complexes displaying selective cytotoxicity under hypoxic conditions, making them potential candidates for targeting tumor regions resistant to conventional therapies. Additionally, these complexes show promising antibacterial and genotoxic effects, suggesting broad therapeutic potential. The biological efficacy is influenced by the metal's coordination environment and oxidation state, which could be crucial for optimizing selectivity and potency in therapeutic applications. The selective cytotoxicity under hypoxic conditions opens new avenues for targeting difficult-to-treat tumours, warranting further optimization for better stability, bioavailability, and targeting mechanisms.

### 3.3. Silver complexes

According to Al-Sulami *et al.* (2023), the antimicrobial efficacy of Ag(i) complexes Ag_1a–c_ was assessed using the agar well diffusion assay. The results demonstrated significant antibacterial and antifungal activities. Ag_1a_ exhibited a zone of inhibition (ZOI) of 21.6 mm against *E. coli*, close to the standard antibiotic gentamicin (27 mm). Ag_1b_ showed substantial activity against *S. mutans* with a ZOI of 26.3 mm. All complexes were effective against fungi such as *C. albicans* and *A. flavus*, indicating their potential as alternative treatments against drug-resistant pathogens.^86^

In their 2020 study, Bharathi *et al.* investigated the biological applications of silver(i) Ag_2a–d_ metallodrugs comprising thiosemicarbazones and naproxen. They evaluated biocompatibility, *in vitro* anti-proliferative activity, and silico interactions with EGFR, VEGFR2, and LOX receptors for each complex, revealing high biocompatibility in normal cells (>80% viability at 100 μM) and selective cytotoxicity against cancer lines, with specific IC_50_ values reported for MCF-7 (2.38 μM), MDA-MB-231 (2.02 μM), and PANC-1 (3.10 μM) for Ag_2a_; MCF-7 (IC_50_ = 1.65 μM), MDA-MB-231 (IC_50_ = 1.91 μM), and PANC-1 (IC_50_ = 2.93 μM) for Ag_2b_; MCF-7 (IC_50_ = 2.15 μM), MDA-MB-231 (IC_50_ = 2.03 μM), and PANC-1 (IC_50_ = 3.41 μM) for Ag_2c_; and MCF-7 (IC_50_ = 6.72 μM) for Ag_2d_, indicating potential for targeted therapy.^87^

Hassan *et al.* found that the Ag(i) complex Ag_3_ exhibited potent antimicrobial activity, with inhibition zones reaching 29 mm against *E. coli* and 28 mm against *C. albicans*. In cytotoxicity assays against breast cancer cells, it demonstrated a low IC_50_ value of 62.36 μg mL^−1^, suggesting strong anticancer potential.^88^

The study by Abdalla *et al.* investigated the antibacterial efficacy of silver(i) complex Ag_4_ derived from a novel hydrazone ligand. Results revealed strong inhibition rates against *Streptococcus pyogenes* (75.51% to 80.34%) and *Escherichia coli* (48.76% to 70.63%) at various concentrations. Notably, irradiated silver(i) complexes exhibited heightened activity compared to non-irradiated ones, hinting at their potential for enhanced antibacterial applications.^89^

The antimicrobial activity of the Ag(i) complex (Ag_5_), studied by Altowyan *et al.*, revealed varied inhibition zone diameters (IZDs) against bacterial and fungal strains. For Gram-positive bacteria, *Staphylococcus aureus* and *Bacillus subtilis*, IZDs were 8 and 9 mm, respectively, while for Gram-negative bacteria, *Escherichia coli* and *Pseudomonas vulgaris*, IZDs were 12 and 15 mm, respectively. The complex demonstrated higher potency against Gram-negative strains, with the lowest minimum inhibitory concentration (MIC) of 625 μg mL^−1^ observed against *P. vulgaris*. It exhibited notable cytotoxicity against colon carcinoma cells, with an IC_50_ of 12.53 μg mL^−1^, indicating high cytotoxic activity compared to the free ligand (IC_50_: 242.92 μg mL^−1^). However, its antioxidant activity was relatively low, with a %D PPH scavenging of 75.18% at 1280 μg mL^−1^ and an IC_50_ of 626.91 μg mL^−1^.^90^

Recent studies have explored the anticancer potential of various silver(i) complexes, especially those involving hydrazide–hydrazones. Notably, Santos *et al.* synthesized a series of complexes (Ag_6a–d_). These complexes demonstrated selective cytotoxicity towards B16F10 metastatic murine melanoma cells, with an IC_50_ around 2 μM, while requiring higher concentrations to affect non-cancerous murine Melan-A melanocyte cells.^91^

Elsayed *et al.* developed a series of metal complexes derived from 3-formyl chromone and benzohydrazide, with the Ag(i) complex containing PPh_3_ (Ag_7_) exhibiting superior cytotoxicity compared to cisplatin against human breast cancer (MDA-MB-231) and ovarian cancer (OVCAR-8) cell lines, with IC_50_ values approximately 1 μM.^92^ Similarly, Ganesh Babu's group investigated a simple Ag(i) complex (Ag_8_), which demonstrated anti-proliferative activity against the MCF-7 cancer cell line with an IC_50_ of 57.45 μM, indicating some efficacy but with relatively high IC_50_ values.^93^

Elsayed *et al.* explored the biological applications of a silver(i) complex (Ag_9_), demonstrating its significant interaction with calf thymus DNA (ctDNA) with a binding constant (*K*_b_) of 4.52 × 10^4^ M^−1^. This complex also exhibited substantial binding affinity towards bovine serum albumin (BSA) with a Stern–Volmer constant (*K*_sv_) of 7.83 × 10^4^ M^−1^. Indicating strong interaction capabilities with biomolecules. Furthermore, *in vitro* studies revealed Ag_9_ potent anticancer activity with IC_50_ values of 8.1 μM against MCF7 breast cancer cells and 11.3 μM against HeLa cervical cancer cells, underscoring its potential as an effective cytotoxic (Table 3).^94^

**Table 3:** Summarizing the antimicrobial (A) and anticancer (B) activities of various Ag(i) complexes

Complex	Target organism/cell line	Activity measure	Reference
**A**			
Ag_1a_	*E. coli*	21.6 mm	86
Ag_1b_	*S. mutans*	26.3 mm	
Ag_3_	*E. coli*	29 mm	88
Ag_4_	*S. pyogenes*	75.51–80.34%	89
Ag_5_	*S. aureus*	8 mm	90
	*B. subtilis*	9 mm	
	*E. coli*	12 mm	
	*P. vulgaris*	15 mm	

**B**			
Ag_2a_	MCF-7	2.38	87
	MDA-MB-231	2.02	
	PANC-1	3.10	
Ag_2b_	MCF-7	1.65	
	MDA-MB-231	1.91	
	PANC-1	2.93	
Ag_2c_	MCF-7	2.15	
	MDA-MB-231	2.03	
	PANC-1	3.41	
Ag_2d_	MCF-7	6.72	
Ag_6a–d_	B16F10 melanoma	∼2	91
Ag_7_	MDA-MB-231	∼1	92
	OVCAR-8	∼1	
Ag_8_	MCF-7	57.45	93
Ag_8_	MCF-7	8.1	94
	HeLa	11.3	

**Fig. 14:**
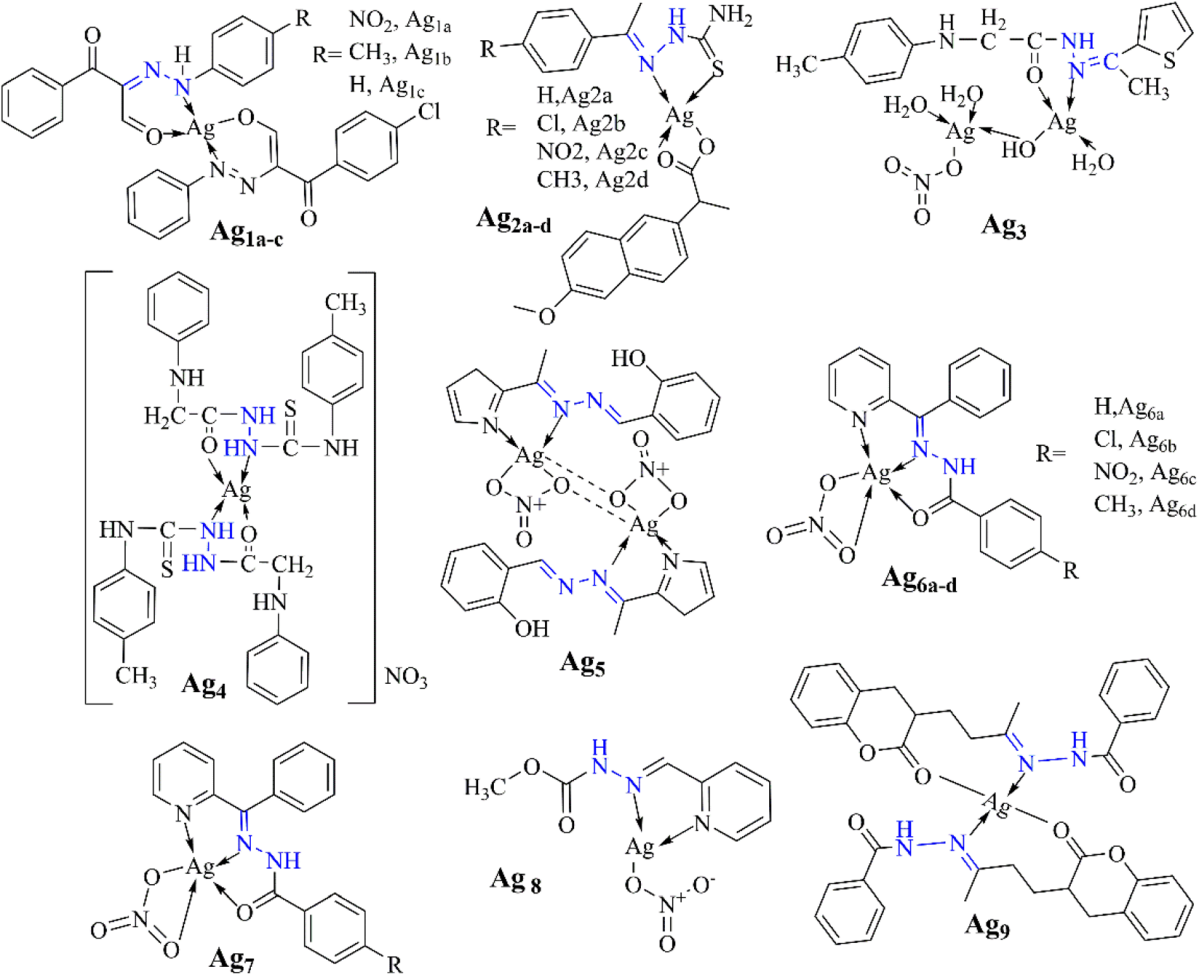
Structure of some silver complexes (Ag_1_–Au_9_).^86–94^

Generally, silver(i) complexes, particularly those involving hydrazone derivatives, have shown broad-spectrum antimicrobial and anticancer potential. Studies have demonstrated their significant antibacterial and antifungal activities, with some complexes, such as Ag_1a_, showing zones of inhibition comparable to standard antibiotics like gentamicin. These complexes also exhibit potent cytotoxicity against cancer cell lines, including MCF-7, MDA-MB-231, and PANC-1, with IC_50_ values in the micromolar range, indicating their selectivity and promise for targeted cancer therapy. Furthermore, the complexes' antimicrobial efficacy, especially against Gram-negative bacteria and low cytotoxicity against normal cells, emphasizes their potential as alternative treatments for drug-resistant infections. In some cases, irradiated silver complexes even showed enhanced antimicrobial activity. The ability of silver(i) complexes to interact with biomolecules, including DNA and serum proteins, highlights their diverse biological activities, suggesting their utility not only in infection control but also in cancer therapy. However, variations in their biological effectiveness, such as lower antioxidant activity, suggest room for optimization to enhance their therapeutic properties. These findings point to further investigation into the structural and coordination characteristics of silver(i) hydrazone complexes to better understand and harness their full potential for medical applications (Fig. 14).

## 4. Conclusion and future outlook

### 4.1. Conclusion

In conclusion, hydrazones and their complexes with coinage metals (copper, silver, and gold) show promising anticancer and antibiotic properties. The coordination with metals enhances the stability and bioactivity of hydrazones, which improves their interactions with cancer cells and pathogenic microorganisms. These complexes exhibit strong anticancer effects through mechanisms such as DNA binding, induction of apoptosis, and cell cycle arrest. Additionally, they demonstrate significant antimicrobial activity against a wide range of bacterial and fungal strains. The unique combination of hydrazone ligands and coinage metals positions these compounds as valuable candidates for developing new antibiotic and anticancer agents.

**Fig. 15:**
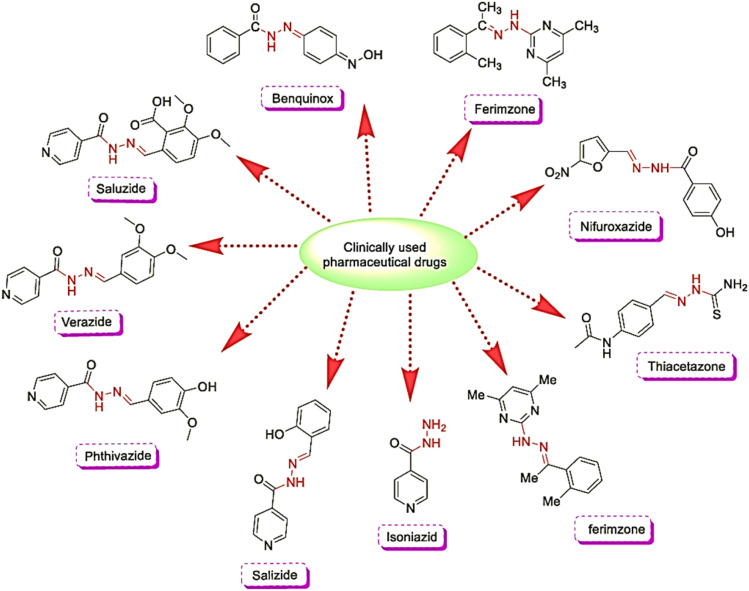
Hydrazone-based commonly prescribed drugs.^95–98^https://doi.org/10.1016/j.mtchem.2020.100349.

More specifically, some notable works also demonstrated the versatility of multi-functionality. For example, the hydrazone-based Zn(ii) complex (ZnAMTC), exhibiting son dynamic therapy (SDT) efficacy against tumors, can be considered proof of concept.^29^ While the current research status of hydrazone-based compounds is well developed up to commercialization (Fig. 15), their coinage metal complexes research status still needs to be revised, which needs further work.

### 4.2. Future outlook

Although the prospect of hydrazone and their coinage metal complexes is bright, further research is needed focusing on the following fields.

➢ Apart from the clinically approved hydrazone-based compounds (see Fig. 15), the real application of hydrazone-based complexes to clinical trials still requires extensive work, including validation of safety, pharmacokinetics, and efficacy.

➢ Further mechanistic studies are still needed to clarify the compounds' interactions with cellular targets and their modes of action.

➢ Accurate assessment requires testing in more advanced models, such as tumor spheroids, animal models, and bacterial infection models.

➢ Additional work is required to optimize these complexes, focusing on stability, bioavailability, metabolic pathways, stability, and reducing toxicity before human trials.

➢ The feasibility of the clinical application of these metal complexes remains uncertain, demanding collaborative work from interdisciplinary fields such as bioinorganic chemistry, medicinal microbiology, and related fields.

## Data availability

The data supporting this study's findings are derived from previously published sources, as cited throughout the article. No new data were created in this study. For further information regarding data sources or to access the datasets discussed, please refer to the original publications in the references list.

## Author contributions

Dessie A., writing the original draft, conceptualization, investigation, editing and detail discussion. Fikre E., Taju S., and Tsegaye B.: were participated in reviewing and editing of the manuscript. Mamo G., Tesfay G., and Ibrahim N. were participated in supervising, reviewing and editing of the manuscript. All authors agree with the final form of a manuscript.

## Conflicts of interest

There are no conflicts to declare.
